# A proteomic view of *Caenorhabditis elegans *caused by short-term hypoxic stress

**DOI:** 10.1186/1477-5956-8-49

**Published:** 2010-09-21

**Authors:** Hualing Li, Changhong Ren, Jinping Shi, Xingyi Hang, Feilong Zhang, Yan Gao, Yonghong Wu, Langlai Xu, Changsheng Chen, Chenggang Zhang

**Affiliations:** 1 Life Science College of Nanjing Agriculture University, Nanjing 210095, China; 2 Beijing Institute of Radiation Medicine, State Key Laboratory of Proteomics, Beijing 100850, China; 3 Medical College of Yangzhou University, Yangzhou 225001, China; 4 Department of Health Statistics, School of Military Preventive Medicine, Fourth Military Medical University, Xi'an 710032, China

## Abstract

**Background:**

The nematode *Caenorhabditis elegans *is both sensitive and tolerant to hypoxic stress, particularly when the evolutionarily conserved hypoxia response pathway HIF-1/EGL-9/VHL is involved. Hypoxia-induced changes in the expression of a number of genes have been analyzed using whole genome microarrays in *C. elegans*, but the changes at the protein level in response to hypoxic stress still remain unclear.

**Results:**

Here, we utilized a quantitative proteomic approach to evaluate changes in the expression patterns of proteins during the early response to hypoxia in *C. elegans*. Two-dimensional difference gel electrophoresis (2D-DIGE) was used to compare the proteomic maps of wild type *C. elegans *strain N2 under a 4-h hypoxia treatment (0.2% oxygen) and under normoxia (control). A subsequent analysis by MALDI-TOF-TOF-MS revealed nineteen protein spots that were differentially expressed. Nine of the protein spots were significantly upregulated, and ten were downregulated upon hypoxic stress. Three of the upregulated proteins were involved in cytoskeletal function (LEV-11, MLC-1, ACT-4), while another three upregulated (ATP-2, ATP-5, VHA-8) were ATP synthases functionally related to energy metabolism. Four ribosomal proteins (RPL-7, RPL-8, RPL-21, RPS-8) were downregulated, indicating a decrease in the level of protein translation upon hypoxic stress. The overexpression of tropomyosin (LEV-11) was further validated by Western blot. In addition, the mutant strain of *lev-11(x12*) also showed a hypoxia-sensitive phenotype in subsequent analyses, confirming the proteomic findings.

**Conclusions:**

Taken together, our data suggest that altered protein expression, structural protein remodeling, and the reduction of translation might play important roles in the early response to oxygen deprivation in *C. elegans*, and this information will help broaden our knowledge on the mechanism of hypoxia response.

## Background

Hypoxic stress can induce apoptosis but also trigger adaptive mechanisms for cell survival. Mammalian cells respond to hypoxia by changes in the expression of numerous genes and proteins to increase anaerobic energy production, protect cells from hypoxic stress, and increase local angiogenesis [1,2]. Recently, the nematode *Caenorhabditis elegans *(*C. elegans*) has been proven to be an valuable model organism for studying the molecular response to hypoxia [3,4]. Although *C. elegans *is sensitive to hypoxic stress, resulting in a phenotype characterized by decreased levels of feeding, movement, and oxygen consumption, it can also survive oxygen tensions between 1% and 0.2% by activating the hypoxia response pathway or anoxic conditions by entering suspended animation. Worms at all stages of development can survive at least one day of anoxia with a viability of 90% [5-7], and *C. elegans *is relatively hypoxia-resistant compared to most mammalian cells [7,8]. Powell Coffman *et al*. reported that the *C. elegans *homolog of the HIF-1 (hypoxia induced factor-1) α subunit is hif-1 and that the HIF-1/EGL-9/VHL pathway is evolutionarily conserved [9]. The most surprising finding was the first globin gene in the genome of *C. elegans *since these small worms were generally thought to rely on diffusion from the environment for gaseous exchange due to their lack of a specialized respiratory or circulatory system [10,11]. Recently, Hoogewijs *et al*. identified 33 putative globin genes by a careful *in silico *analysis of the genome of *C. elegans *[12,13]. All of these globins are expressed, and they show a wide diversity in their gene structures and amino acid sequences. Ten globins are responsive to oxygen deprivation through interactions with HIF-1 and DAF-16. The explanations for the large number of globins and their functions in this tiny worm remain a mystery. In addition, the determinants of the hypoxia response and survival in *C. elegans *are still not completely understood. Therefore, these mechanisms should be studied to gain more knowledge on oxygen-deprivation sensing and survival in *C. elegans*.

The response of *C. elegans *to hypoxia, including its behavioral and physiological issues, has been well studied [14-16]. More recently, genomics studies have identified a comprehensive set of hypoxia response genes in *C. elegans *[17-19]. For example, Shen *et al*. compared hypoxia-induced changes in mRNA expression in wild type, *hif-1*-deficient, and *vhl-1*-deficient *C. elegans *strains using whole genome microarrays [20], which resulted in the identification of 110 hypoxia-regulated gene expression changes after 4 h under hypoxia. Because the expression of the proteins are directly related to cellular functions [21], other reports have examined the proteome-wide alterations during hypoxia in mammalian cells [22,23], zebrafish [24], rainbow trout [25], shrimp [26] and others. As frequently mentioned, analysis of gross proteomic changes, which would complement the available mRNA data, might be necessary to achieve a better insight into the hypoxia response. A proteomic study on hypoxia in *C. elegans*, however, has not yet been performed.

In *C. elegans*, proteomic approaches are becoming part of the essential toolbox for the study of gene function [27]. For example, these studies have been applied to the investigation of differentially expressed proteins during postembryonic development [28] or when living at different temperatures [29]. Mawuenyega *et al*. also analyzed the worm proteome by 2D gel electrophoresis liquid chromatography (2D-LC) combined with electrospray ionization tandem mass spectrometry (ESI-MS/MS) and identified a total of 1,616 proteins, including 110 secreted/targeted proteins and 242 transmembrane proteins [30]. Quantitative proteomics can efficiently provide accurate and reproducible differential expression values for proteins [31,32]. Two-dimensional difference gel electrophoresis (2D-DIGE) has been successfully used for the proteomic analysis of various biological and medical subjects, such as the identification of differentially expressed proteins in *Escherichia coli *[33]*, Saccharomyces cerevisiae *[34], mouse [35] and human hepatocellular carcinoma [36]. Using 2D-DIGE, Tabuse *et al*. compared the proteome maps from all six developmental stages, embryonic, L1-L4 larva1, and adult, to examine the expression profiles of 165 proteins during *C. elegans *development [37].

To better understand the molecular mechanisms underlying the response to hypoxic stress at the protein level in *C. elegans*, a 2D-DIGE and mass spectrometry (MS) based approach was used to determine the overall profile of protein expression during the early response to hypoxia. Differentially expressed protein spots were identified, and the proteins of interest were validated by Western blot. Further study of one of the hypoxia-induced proteins expressed at a high level was performed by using a mutant strain obtained from the Caenorhabditis Genetics Center (CGC). Consistent with our proteomic findings, the *lev-11(x12*) mutant strain had a higher death rate than the wild type strain N2 under hypoxic stress. To the best of our knowledge, this study is the first to apply a proteomic approach to screen differentially expressed proteins in response to hypoxia in *C. elegans*, and the results may provide new clues to understand the mechanism of hypoxia response better.

## Results

### 2D-DIGE-based proteomic analysis

**Figure 1:**
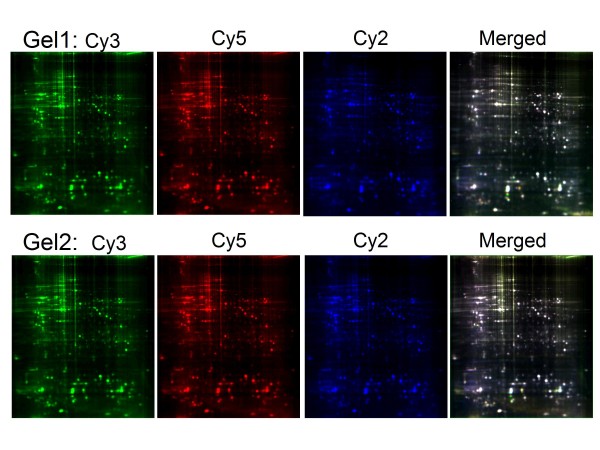
**DIGE maps of the *C. elegans *wild type (N2) after 4 h hypoxia**. In GEL1 (top panel), the control lysate was labeled with Cy3 (green), and the lysate from 4 h hypoxia group was labeled with Cy5 (red). In GEL2 (bottom panel), reverse labeling was used so that the control was labeled with Cy5 (red) and the hypoxia group was labeled with Cy3 (green). For normalization in both gels, a mixture containing equal parts of the control and hypoxia groups was labeled with Cy2 (blue). The three Cy-dye labeled samples were co-separated on one gel, and the merged gel was created by the overlay of the three images. In total, 1,231 spots were matched from each of the fluorescent gels.

To determine the proteins that were differentially expressed proteins during the early stage of the hypoxia response, a proteomic study using 2D-DIGE followed by MALDI-TOF-TOF-MS was performed on the wild type strain N2 of *C. elegans *under hypoxia or normoxia (control) for 4 h. The labeled samples were separated, and the fluorescent images were obtained (Figure 1). The lysate extracted from the hypoxia and control groups were labeled with either Cy5 (Figure 1, top panel) or Cy3 (Figure 1, bottom panel) to enable all comparisons and eliminate any dye-labeling bias, while equal amounts of protein from the sample pairs were pooled together as an internal standard and labeled with Cy2. The normalized ratio of the protein expression level for each spot was calculated relative to the internal standard, and inter-gel spot matching was performed using the DeCyder software. The incorporation of this internal standard on each gel has been reported to improve the accuracy of spot matching and the reliability of the quantitative comparison of the spots between gels [32]. Therefore, this method was sufficient to enable us running two DIGE-gels at the same time. Bioinformatic analyses of the images detected approximately 1,308 significantly altered spots in GEL1, 1,307 spots in GEL2, and a total of 1,231 spots in both gels. The matching ratio of the protein spots between the two gels was approximately 94.1%, indicating that DIGE-based proteomic analysis is an appropriate method to study hypoxia in *C. elegans*. Many of the protein spots are similar between the two experimental conditions, likely representing a L4-stage wild type strain-related proteomic profile.

### Identification of differentially expressed proteins

**Figure 2:**
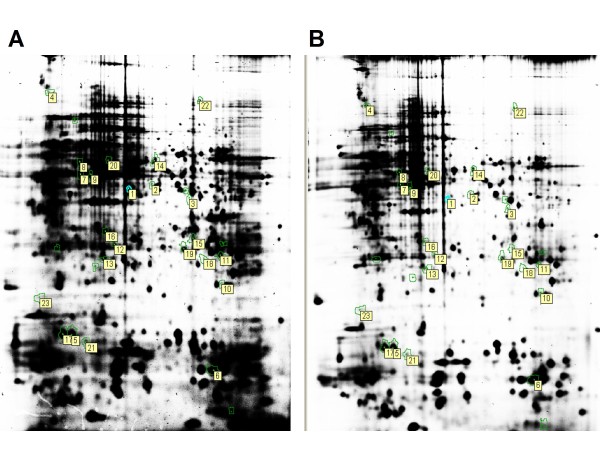
**The 2D-DIGE protein map**. The number and positions of 23 selected spots (ratio_hypoxia/control _≥ 1.5 for upregulated spots and ≤ -1.5 for downregulated spots, *P *< 0.05) labeled from 1 to 23 within the brackets for identification by MALDI-TOF/TOF-MS are shown. (A) control; (B) 4 h hypoxia. A total of 19 proteins were successfully identified.

Among the 1,231 matched protein spots that satisfied the arbitrary parameters defined as the relative expression ratio_hypoxia/control _≥ 1.5 for upregulated spots and ≤ -1.5 for downregulated spots, 23 protein spots displaying significant changes (*P *< 0.05) were selected (Figure 2). Each spot of interest was excised from preparative gel and analyzed by MALDI-TOF/TOF-MS after in-gel tryptic digestion. The combined spectra were searched against the Swiss-Prot database of *C. elegans *using a Mascot engine, which resulted in the successful identification of nineteen spots. The protein name, accession number, theoretical molecular weight, *P *value and *pI *values, spot number and score are shown in Table 1. Under hypoxia, nine of these proteins were significantly upregulated, and ten were downregulated. These data represent the first description of the regulatory expression profiles of *C. elegans *during early hypoxia. The upregulated proteins were associated with structural proteins, ATP synthesis and energy metabolism, whereas the downregulated proteins were mainly associated with ribosomal proteins.

**Table 1:** List of differently expressed proteins in *C. elegans *wild type strain N2 with hypoxia treatment for 4 h

Spot No.	Protein name	Score	Accession number	Theoretical mass (Da)	Theoretical *pI*	*P *value
**Up-regulated proteins (totally 9)**						
1	Isoform a of tropomyosin isoforms a/b/d/f. lev-11	94	Q22866-1	32984	4.66	0.037
2	Protein F27D4.5, confirmed by transcript evidence.tag-173	177	Q93619	40027	6.22	0.028
3	Isoform a of probable arginine kinase. F46H5.3	152	Q10454-1	44140	6.84	0.002
5	Myosin regulatory light chain 1. mlc-1	496	P19625	18605	5.06	0.004
6	Fatty-acid and retinol-binding protein 1.far-1	528	P34382	20097	6.98	0.031
7	Actin protein 4, isoform c. act-4	530	Q6A8K1	40400	5.56	0.021
8	ATP synthase subunit beta, mitochondrial. atp-2	401	P46561	57491	5.52	0.045
10	Protein C06H2.1, confirmed by transcript evidence.atp-5	245	Q17763	21784	6.67	0.042
11	Vacuolar h Atpase protein 8.vha-8	153	Q95X44	25570	6.78	0.048
**Down-regulated proteins (totally 10)**						
12	Protein ZK593.9, partially confirmed by transcript evidence	66	Q23538	595560	9.07	0.032
13	Isoform b of probable arginine kinase. F46H5.3	83	Q10454-2	39966	6.17	0.017
14	Isoform a of muscle M-line assembly protein. unc-89	60	O01761-2	731233	5.35	0.001
15	60S ribosomal protein L7. rpl-7	80	O01802	28114	10.16	0.022
16	60S ribosomal protein L8. rpl-8	72	Q9XVF7	28186	11.07	0.040
17	Myosin regulatory light chain 2. mlc-2	493	P19626	18591	5.06	0.034
18	40S ribosomal protein S8. rps-8	133	P48156	23736	10.56	0.027
19	Heat shock 70 kDa protein A. hsp-1	95	P09446	69680	5.44	0.034
21	Protein Y54E5A.5, confirmed by transcript evidence	152	Q9XWK2	17426	5.24	0.030
23	60S ribosomal protein L21. rpl-21	232	P34334	18299	11.15	0.027

### Confirmation of protein expression by Western blot

**Figure 3:**
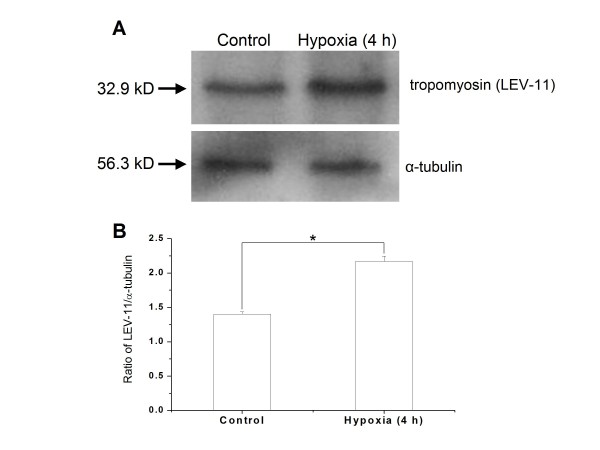
**Validation of the upregulation of the tropomyosin (32.9 kDa) protein in wild type N2 after 4 h hypoxia treatment**. (A) Western blot analysis showing the upregulation of tropomyosin protein in wild type N2 after 4 h hypoxia treatment compared with the control. The protein α-tubulin (56.3 kDa) was used as an internal control. (B) Image analysis showing that the expression of tropomyosin (LEV-11) is significantly upregulated at 4 h hypoxia compared with the control (*P *= 0.0069). The expression pattern of LEV-11 was consistent with the quantitative proteomic results.

To evaluate the performance of the quantitative proteomic approach used in this study, the worm protein tropomyosin (LEV-11), identified as the most highly induced protein by hypoxia, was further examined by Western blot. Tropomyosin (LEV-11) was significantly upregulated in the 4-h hypoxia treatment samples when compared with the controls (*P *= 0.0069, Figure 3). The agreement between the 2D-DIGE and Western blot results basically validates this proteomic approach.

### Hypoxia-induced death rates of the tropomyosin gene mutant strain *lev-11(x12*) and wild type *C*. *elegans*

**Figure 4:**
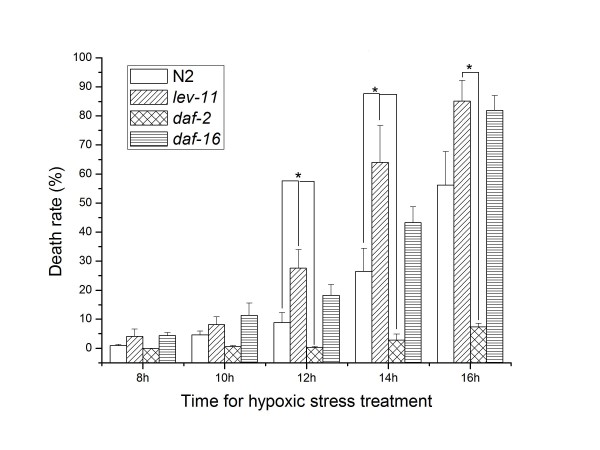
**Death rate of the *lev-11*(*x12*), *daf-2 *(hypoxia-resistant) and *daf-16 *(hypoxia-sensitive) mutants and that of the wild type animals (N2) exposed to 8, 10, 12, 14 and 16 h of hypoxia (0.2% oxygen) and after a 24-h recovery (21% oxygen)**. The death rate of the wild type N2 strain and *lev-11*(*x12*) mutant gradually increased as the time under hypoxic conditions increased. The *daf-2 *mutants were highly resistant to hypoxia, whereas the *daf-16 *mutants were highly sensitive to hypoxia. The death rate was significantly different when the wild type animals (8.85% ± 3.50, 26.44% ± 7.96) were compared with the *lev-11 (x12*) strain (27.64% ± 6.30, 64.06% ± 12.00) after 12 h and 14 h of hypoxia treatment, respectively (*P *= 0.0183, *P = *0.0401). After 12 h of hypoxia, the death rate of the *lev-11 (x12*) strain was higher than that of the *daf-16 *mutant. The results are displayed as means ± standard deviation, n > 3, *: *P *< 0.05.

To determine the function of tropomyosin (LEV-11), the survival ability of the L4 stage of the wild type and the mutants *lev-11(x12)*, *daf-2 *(hypoxia-resistant), and *daf-16 *(hypoxia-sensitive) were examined under hypoxic conditions (26°C, 0.2% O_2_). The death rate of the wild type N2 strain and *lev-11*(*x12*) mutant gradually increased as the time under hypoxic conditions increased as well as after a 24-h recovery (Figure 4). The *daf-2 *mutants were highly resistant to hypoxia, while the *daf-16 *mutants were highly sensitive to hypoxia. The death rate was significantly different when the wild type animals (8.85% ± 3.50, 26.44% ± 7.96, n > 3) were compared with the *lev-11 (x12*) strain (27.64% ± 6.30, 64.06% ± 12.00, n > 3) after 12 h and 14 h of hypoxia treatment (*P *= 0.0183, *P *= 0.0401), respectively. When compared with the wild type worms, the *lev-11 (x12*) mutants have a higher death rate and are more sensitive to oxygen deprivation. After 12 h of hypoxia, the death rate of the *lev-11 (x12*) strain was higher than that of the *daf-16 *mutant. These data further support the proteomic data presented here and suggest that the protein tropomyosin (LEV-11) has an important role in the early hypoxia response and survival for *C. elegans*.

### GO analysis

A gene ontology (GO) classification was performed to analyze the distribution of the differentially expressed proteins identified in this study within the different biological process categories (Table 2). The largest protein set belongs to the functional group of proteins involved in larval development (*P *= 6.54E-06). Other proteins are involved in the positive regulation of growth, ATP metabolic process, purine ribonucleotide binding, hydrogen transport, and proton transport, with significant *P*-values. These biological processes are all consistent with changes made in response to stress conditions. These classifications provide clues to understand better the proteomic expression changes in the nematode *in vivo *during the early response to hypoxia.

**Table 2:** Gene ontology analysis of differently expressed proteins identified by MALDI-TOF/TOF MS

GO ID	GO term	Number of proteins	Fold enrichment	*P *value
GO:0002119	larval development	11 (lev-11, rpl-8, rps-8, rpl-21, act-4, mlc-2, rpl-7, hsp-1, atp-2, atp-5, mlc-1)	3.71	5.60E-05
GO:0002164	larval development	11 (lev-11, rpl-8, rps-8, rpl-21, act-4, mlc-2, rpl-7, hsp-1, atp-2, atp-5, mlc-1)	3.71	5.67E-05
GO:0015986	ATP synthesis coupled proton transport	3 (vha-8, atp-2, atp-5)	31.33	3.50E-03
GO:0046034	ATP metabolic process	3 (vha-8, atp-2, atp-5)	29.37	3.98E-03
GO:0006818	hydrogen transport	3 (vha-8, atp-2, atp-5)	23.49	6.15E-03
GO:0015992	proton transport	3 (vha-8, atp-2, atp-5)	23.49	6.15E-03
GO:0016820	hydrolase activity, acting on acid anhydrides, catalyzing transmembrane movement of substances	3 (vha-8, atp-2, atp-5)	13.50	1.68E-02
GO:0015399	primary active transmembrane transporter activity	3 (vha-8, atp-2, atp-5)	13.28	1.73E-02
GO:0051188	cofactor biosynthetic process	3 (vha-8, atp-2, atp-5)	12.59	2.04E-02
GO:0030554	adenyl nucleotide binding	6 (unc-89, act-4, hsp-1, atp-2, atp-5, ZK593.9)	3.07	2.22E-02
GO:0006732	coenzyme metabolic process	3 (vha-8, atp-2, atp-5)	11.37	2.46E-02
GO:0009165	nucleotide biosynthetic process	3 (vha-8, atp-2, atp-5)	11.37	2.46E-02
GO:0032555	purine ribonucleotide binding	6 (unc-89¬, act-4, hsp-1, atp-2, atp-5, ZK593.9)	2.79	3.26E-02
GO:0045927	positive regulation of growth	7 (far-1, mlc-1, atp-2, atp-5, rpl-7, rpl-8, tag-173)	2.45	3.71E-02
GO:0055086	nucleobase, nucleoside and nucleotide metabolic process	3 (vha-8, atp-2, atp-5)	8.49	4.21E-02
GO:0006796	phosphate metabolic process	5 (unc-89,ZK593.9, vha-8, atp-2, atp-5)	3.31	4.61E-02

## Discussion

*C. elegans *is a model organism for research in the fields of developmental biology, molecular biology, neurobiology and toxicology [38-40]. Here, we showed that *C. elegans *can also be used as a model organism for hypoxia studies. Using 2D-DIGE followed by protein identification through mass spectroscopy, nineteen proteins that responded during the early stage of hypoxia exposure were successfully identified. Although only a small number of proteins was identified, a precise evaluation of the results presented here still needs to be performed.

It is especially important for using the proteomic approach to determine the differently expressed protein spots from multiple independent experiments. Here we ran three gels for each DIGE analysis on one batch of *C. elegans*, and we totally used three batches of *C. elegans *as independent experiments to meet the standard for biological replicates. In addition, the quantitative proteomic method, DIGE, was used in this study. Briefly, samples were reverse-labeled (cy3 or cy5) to enable all comparisons and eliminate any dye-labeling bias. Samples were also mixed together and run on the same gels with an equal amount of the cy2-labeled standard. Cy2 was used as a standard on all gels to aid image matching and cross-gel statistical analyses. The use of this internal standard in all 2-DE gels greatly improved the accuracy of spot matching and the reliability of protein quantification. Therefore, this method was sufficient to enable us running two DIGE-gels at the same time, and the Decyder software was used to analyze the data. Protein spots that were differentially expressed in hypoxia-treated and control groups (ratio: hypoxia/control ≥ 1.5 or hypoxia/control ≤ -1.5, *P *< 0.05) were marked. Protein samples were extracted from the nematodes at least eight times, and the extracted were mixed together. All these operations are important to improve the fidelity of the protein spots identified in this study.

### Structural proteins involved in cytoskeletal organization

Proteins contained several structural proteins, including actin (ACT-4), the myosin regulatory light chain (MLC) and tropomyosin (LEV-11), were upregulated during early hypoxic stress in the L4 stage. These proteins are typical structural proteins that are ubiquitously expressed in all eukaryotic cells and that play essential roles in myofibril assembly and muscle contraction. *C. elegans *has only two main muscle tissues, which are the body wall for locomotion and the pharynx for feeding. The actin cytoskeleton is important for many cellular functions, including cell motility, structure and integrity. *C. elegans *has four actin genes that are highly homologous [41]. Isoforms d (act-4) and b (act-2) have the most similar amino acid sequences. In our study, actin isoform d was upregulated after hypoxic stress. Homologs of the actin isoform b were upregulated after long-term anoxia in the rainbow trout hypodermal fibroblast cell-line [25] and after a one- to two-week exposure to hypoxia in the pulmonary arterial smooth muscle cells of rats [42]. In human umbilical vein endothelial cells at lower oxygen concentrations [43], however, actin isoform b was downregulated. These contrasting responses of actin to hypoxia might be the result of different stages and experimental conditions as well as suggest that the actin isoforms might have different roles in the response to hypoxic stress in different organisms.

Mlc-1 and Mlc-2 both encode a muscle regulatory myosin light chain that is involved in L1 larval viability, elongation, muscle development of the pharynx and body wall, locomotion and growth [44]. The role of MLC in the regulation of muscle cell contraction is well characterized [45], but its function during hypoxic stress is not completely understood. In the current study, hypoxia induced the synthesis of MLC-1 but decreased the amount of MLC-2. MLC-1 and MLC-2 are nearly identical with the exception of a single conservative amino acid substitution [44]. This difference in gene structure might reflect the differences in their expression patterns.

Unc-89 encodes isoform a of the muscle M-line assembly protein. UNC-89 is required for the proper organization of A bands in striated muscle and thus for normal locomotion, pharyngeal muscle contraction, and body size [46]. UNC-89 was downregulated under these hypoxic conditions, which suggests that the striated muscle structure becomes disorganized after hypoxia treatment. This notion needs to be further studied.

Interestingly, the most highly upregulated protein was tropomyosin, which was confirmed by western blot. The protein LEV-11 (spot #1) is a major component of the contractile apparatus of the muscle cells that maintain cell cytoarchitecture. LEV-11 is associated with the actin thin filament and plays a regulatory role in the organization of actin microfilaments and stress fibers. LEV-11 transmits structural changes along the thin filament during the regulatory process. The effect of increased calcium levels could be transferred to actin molecules [47,48]. The increased synthesis of tropomyosin allows *C. elegans *to maintain its shape and cell adherence. The hypoxia-induced synthesis of tropomyosin has also been reported in pulmonary arterial myocytes [49], Which is consistent with our data. Lev-11 is orthologous to human tropomyosin 1 (TPM1), in which mutations lead to familial hypertrophic cardiomyopathy [50]. *C. elegans *with the *lev-11 *gene mutant were paralyzed together with abnormal muscle filament assembly [51]. To further analyze the hypoxia response of the *lev*-*11 *gene in nematode, the death rate was compared among *lev*-*11 *(*x12*), *daf-2, daf-16 *mutants and wild type worms. To the best of our knowledge, our results are the first to demonstrate that the *lev*-*11 *(*x12*) mutant displays a hypoxia-sensitive phenotype, which supports the idea that LEV-11 plays a role in the hypoxia response and survival in *C. elegans*.

Therefore, we hypothesized that these cytoskeletal changes have an important role in the early hypoxia response. We propose that the synthesis of structural proteins contributes to the maintenance of the shape and function of the nematodes. These proteins are believed to be responsible for helping the worms adjust to an oxygen-deprived environment.

### Proteins related to energy production

The proteins ATP-2, ATP-5 and VHA-8 that were identified in this study are associated with energy and metabolism. Their altered expression levels are consistent with the altered energy metabolism seen under hypoxic conditions. *ATP-2 *encodes the beta subunit of the soluble catalytic F1 portion of ATP synthase (mitochondrial respiratory chain complex V) [52]. ATP-5 encodes subunit d of mitochondrial F1/F0-ATP synthase, and vha-8 encodes an ortholog of subunit E of the cytoplasmic (V1) domain of vacuolar proton-translocating ATPase (V-ATPase). These enzymes are involved in aerobic respiration, which is the most efficient metabolic energy pathway. Under normoxia, most energy is produced this way. As a result of the lack of oxygen after hypoxia treatment, the upregulation of these enzymes likely reflects severe energy deficits in these worms, and these enzymes would be quickly activated to produce energy under early hypoxic stress.

F46H5.3 encodes arginine kinase (AK), which is a member of the phosphagen (guanidino) kinase family of highly conserved enzymes that catalyze the reversible transfer of phosphate from a phosphorylated guanidine (~NH-CN_2_H_4_^+^) substrate to ADP to satisfy short-term ATP requirements [53]. AK has significant sequence similarity to creatine kinase and likely serves the same function in the muscle of *C. elegans *as creatine kinase does in mammalian cells [54]. AK isoform a was upregulated, while isoform b was downregulated. The upregulation of AK isoform a suggests an alternative method for the production of ATP to adapt to hypoxic conditions at an early stage. The substrate concentration decreased as the time of the hypoxia treatment increased, and this reaction was gradually reduced over time.

Far-1 encodes the fatty acid and retinol binding protein, which may interfere with intercellular lipid signaling to manipulate the defense reactions of the host or to acquire essential lipids for the nematode [55]. Far-1 was upregulated after the hypoxia treatment, suggesting that an increase of FAR-1 could help the worms produce energy from lipids.

The proteins related to energy and metabolism and discussed here can also be induced by other stress conditions and appear to be important for survival during various stress conditions. Taken together, these observations suggest that energy is important for the maintenance of hypoxic relaxation. To maintain a balance of ATP demand and supply, various pathways should be activated to produce energy. In this study, however, the enzymes involved in anaerobic pathways were not upregulated, as has been seen in other studies [25,56]. This contrast may be related to the short duration of the hypoxic stress, and after long-term hypoxia exposure, glucose might become one of the main fuel sources.

### Ribosomal proteins

Among the ten down-regulated proteins, four are the ribosomal proteins RPL-7, RPL-8, RPS-8, and RPL-21. RPL-7 and RPL-8 encode the large ribosomal subunit L7 protein that has been reported by mass RNAi assays to be required for embryonic viability and normal, rapid growth [57]. RPS-8 encodes the small ribosomal subunit S8 protein, which is predicted to function in protein biosynthesis. RPS-8 activity is required for germline development and the overall health of *C. elegans *[58]. RPL-21 encodes the large ribosomal subunit L21 protein [59], involved in translation in the ribosomal machine. In our study, all four ribosomal proteins were downregulated at the protein level after hypoxia treatment, suggesting that protein synthesis was suppressed in *C. elegans *during hypoxia. The inhibition of protein synthesis and the conservation of energy is advantageous for hypoxic worms because a decreased translation rate will consequently reduce oxygen consumption. Reduced oxygen consumption by translational arrest has been reported to be a logical and established mechanism for the reduction of cellular injury during, but not after, hypoxia [60-62]. Anderson *et al*. also reported that *C. elegans *could survive hypoxia by the inactivation of aminoacyl-tRNA synthetases, enzymes essential for protein translation [63]. Consistent with a reduction in protein synthesis, our data suggest that the downregulation of these ribosomal proteins may contribute to a hypoxia-mediated global translation attenuation in *C. elegans*.

### Chaperones

Heat shock proteins (HSP) have been reported to be induced by many types of stress. In fact, HSP70 and HSP90 family proteins are induced by hypoxia in various systems [64-67]. The HSP proteins are also chaperone proteins that can assist in the folding of proteins. Overexpression of individual molecular chaperones in *C. elegans *has been shown to extend the life span [68]. There are at least nine genes in the HSP70 family of *C. elegans *[69]. In this study, HSP-1, encoding heat shock 70 kDa protein A, was downregulated in response to short-term hypoxia. This observation suggests that the amount of newly synthesized protein could be partially reduced by increasing the amount of unfolded proteins.

### Other proteins

After a short-term exposure to hypoxia, the protein F27D4.5 was upregulated, and the proteins ZK593.9 and Y54E5A.5 were downregulated, which was confirmed by transcript alterations. There are no clear descriptions of the molecular functions of these proteins to date. According to the GO term annotation, F27D4.5 is involved in the positive regulation of growth, and ZK593.9 is related to proteins involved in adenyl nucleotide binding, purine ribonucleotide binding, and the phosphate metabolic process. Further studies on these proteins may help understand their functions under hypoxic stress in *C. elegans*.

Reactive oxygen species (ROS) have been reported to be released during hypoxia, which may contribute to the stabilization of HIF-1α [70,71]. Some hypoxia-related proteins (e.g., globins, HIF-1, ROS), however, were not identified in this study either because their expression level was too low to be detected or there was no significant difference at this time point. The limitations of this approach should also be considered. In addition, none of the differentially expressed proteins had a connection to the HIF pathway. Although there may be a connection between HIF pathway and ATP production and consumption, more studies should be performed to confirm that the HIF pathway is not involved early response to hypoxia and to better understand the role of HIF-1 in the context of temporal expression.

A comparison between hypoxia-induced mRNAs and our proteomic results was performed, but no overlap was found. The low correlation between the levels of mRNA and protein, with the activity of the protein often controlled by various post-translational modifications instead, has become widely accepted [72,73]. Shen *et al*. found that some metabolic enzymes, such as PYC-1, F14B4.2 hexokinase and R05F9.6 phospholglucomutase, were induced by hypoxia, which would induce the production of ATP [20]. In this study, the proteins ATP-2, ATP-5 and VHA-8, which are associated with energy and metabolism, had altered expression levels. Our results showed that three ribosomal proteins were downregulated during hypoxic stress. This observation is closely related to that reported by Mabon, who showed that the inhibition of many different translation machinery proteins can protect from hypoxic injury [17]. Therefore, there are similarities between our proteomic data and the mRNA expression data of others that might represent an interesting correlation.

Scott *et al*. showed that wild type *C. elegans *became immobile after exposure to a severe hypoxic environment (< 0.3% oxygen) but fully recovered when returned to normoxia within 4 h [14]. After 4 h, however, permanent behavioral deficits and cellular death ensue, and after a 22-h hypoxic incubation, > 99% of wild type animals are dead. These deaths are not the result of programmed cell death [74]. In most tissues during ischemia-reperfusion, an initial response has been observed within the first hours (0-4 h), followed by a late response that occurred after approximately 12-24 h [75,76]. Based on these data, the length of hypoxia exposure was selected as 4 h. In addition, L4 stage worms were used because they did not carry embryos yet. Worms that contain eggs might be particularly vulnerable to hypoxic injury and thereby alter the hypoxic sensitivity of the whole organism. The last larval stage is suitable for investigating the effects of hypoxia on the protein expression level of only that generation. The *C. elegans *experiments were usually performed between 15-25°C. Dasgupta *et al*. reported that hypoxia at temperature higher than the normal physiological temperature can still induce a protective response [77], therefore the temperature for hypoxia stress was selected as 26°C in our experiment. This study represents the first proteomic analysis to investigate the altered proteins during a 4-h exposure to hypoxia in L4 stage wild type *C. elegans *at 26°C compared with a control group exposed to normoxic conditions.

The use of animal model systems is widely accepted as being important to understand the response to oxygen deprivation, which is involved in many human health issues, such as cancer, stroke, and cardiac failure. Given that the objective of this study was to gain a better understanding of oxygen-deprivation response and survival mechanisms, our data have provided a proteomic landscape of the molecular response to short-term hypoxia exposure in *C. elegans*. The reorganization of cytoskeletal structural proteins is important for adaptation to hypoxic conditions. The preservation of the cytoskeleton and shape of cells in the presence of hypoxia would allow the worms to maintain its vital need to exchange oxygen. The synthesis of several proteins is important to maintain cell function and integrity. On the other hand, we present new evidence that translation arrest during hypoxia is induced by a downregulation of ribosomal proteins in *C. elegans*. This observation will help to understand the mechanisms involved in hypoxia adaptation better. Given the evolutionarily conserved functions in *C. elegans *(including humans), these data will broaden our understanding of how cellular remodeling might occur during hypoxia and also provide new insights into the hypoxia stress response and survival of *C. elegans *at the protein level. Worm cells appear to cope well during early stage hypoxia. Studies on the protein expression profiles in response to different times of hypoxia exposure will be interesting, and the resulting data obtained in *C. elegans *might be applicable to mammalian cells.

## Conclusions

Taken together, our data suggest that altered protein expression, structural protein remodeling, and the reduction of translation might play important roles in the early response to oxygen deprivation in *C. elegans*, and this information will help broaden our knowledge on the mechanism of hypoxia response.

## Methods

### Synchronized culture and hypoxic treatment of *C. elegans*

The wild type strain N2 (obtained from CGC) of *C. elegans *was cultured at 20°C on 9-cm nematode growth medium agar plates seeded with *E. coli *OP50 as described by Brenner [78]. After washing off the adult worms with M9 buffer (3 g/l KH_2_PO_4_, 6 g/l Na_2_HPO_4_, 5 g/l NaCl, 10 mM MgSO_4_) from the plates, embryos were obtained by dissolving gravid animals with alkaline hypochlorite (0.25 M NaOH, 1.2% NaOCl). Briefly, the pellet of worms (~0.5 ml) was resuspended and dissolved in 5 ml of an alkaline hypochlorite solution with frequent agitation. After centrifugation, the embryos that were released from the worms were washed twice with M9 buffer to remove the carcasses and then incubated at 20°C for a further 16 h to allow hatching. These worms have six development stages: embryo, L1-L4 larvae, and adult. Newly hatched L1 larvae were collected, seeded on fresh plates and incubated to the L4 larval stage. The L4 stage was selected for this study because it cannot carry embryos yet. The synchronized, cultured L4 worms were quickly harvested into a tube with M9 buffer and washed three times. The hypoxia group was incubated for 4 h in a sealed hypoxia chamber with a constant gas flow (95% N_2_, 5% CO_2_) at 26°C, and the oxygen level set to 0.2%, which was monitored by an oxygen probe. The control group was incubated for 4 h in a sealed chamber with a constant gas flow (95% N_2_, 5% CO_2_) at 26°C and the oxygen level set to normal levels (21% O_2_). After the 4 -h incubation, the worms were removed from the chambers and stored in liquid nitrogen before use.

### Sample preparation

After washing with M9 buffer and distilled water, the worms were resuspended in an equal volume of lysis buffer (7 M urea, 2 M thiourea, 4% w/v CHAPS, 30 mM Tris-HCl, pH 8.5) with a protease inhibitor cocktail, frozen in liquid nitrogen, and then ground into powder with a chilled mortar and pestle. The homogenates were sonicated on ice briefly and then centrifuged at 13,000 rpm for 30 min at 4°C. The supernatants were removed and used as the protein samples. Protein samples were extracted from the nematodes at least eight times, and the extracted were mixed together. Protein concentrations were determined using the 2D Quant Kit (GE Healthcare) according to the manufacturer's protocol. The samples were stored at -80°C until use.

### Two-dimensional difference gel electrophoresis (2D-DIGE)

The pH of each protein sample was adjusted to 8.5 with 50 mM NaOH, and the final concentration was adjusted to 5 mg/ml with lysis buffer. Equal amounts of protein from the sample pairs were pooled together as an internal standard. The hypoxia-treated and control samples were randomly labeled with Cy3 or Cy5, whereas the internal standards were labeled with Cy2 using 400 pmol of fluorochrome/50 μg of protein (GE Healthcare). Labeling was performed for 30 min on ice in the dark. The reactions were terminated by adding 1 μl of 10 mM lysine for 10 min on ice in the dark.

After 50 μg each of the Cy3- and Cy5-labeled samples were combined, they were then mixed with 50 μg of the Cy2-labeled internal standard. An equal volume of 2× sample buffer (7 M urea, 2 M thiourea, 4% CHAPS, 1% Bio-Lyte, pH 3-10, 20 mg/ml DTT) was added to the sample, and the total volume was increased to 410 μl with rehydration buffer (7 M urea, 2 M thiourea, 4% CHAPS, 0.5% Bio-Lyte, 10 mg/ml DTT). The samples were actively rehydrated into 18-cm pH 3-10 IPG strips at 17°C for 12 h using an Ettan™ IPGphor IEF System (GE Healthcare). Isoelectric focusing was performed for a total of 80 kV-h (ramped to 250 V in 30 min, held at 1000 V for 1 h, ramped to 10,000 V in 5 h, and held at 10,000 V for 60 kV-h). The IPG strips were equilibrated in equilibration buffer (6 M urea, 2% SDS, 50 mM Tris-HCl, pH 8.8, 30% glycerol) supplemented with 0.5% DTT for 15 min at room temperature, followed with 4.5% iodoacetamide in equilibration buffer for another 15-min incubation at room temperature.

The IPG strips were placed on the top of a 12% homogeneous polyacrylamide gel that had been precast with low fluorescence glass plates using an Ettan DALT twelve gel caster. The second dimension SDS-PAGE was carried out using the Ettan DALT II (GE Healthcare). After two-dimensional electrophoresis (2DE), the gels were scanned on the Typhoon 9410 scanner with Ettan DALT gel alignment guides using excitation/emission wavelengths specific for Cy2 (488/520 nm), Cy3 (532/580 nm), and Cy5 (633/670 nm). The intensity was adjusted to ensure that the maximum volume of each image was within 60,000-90,000.

The gel-to-gel spot matching and statistical analyses of the protein abundances among the samples were carried out with the DeCyder 5.0 BVA (biological variation analysis, GE Healthcare) according to the manufacturer's protocol. Statistically significant differences were calculated by a paired Student's *t*-test, and the significance level was set at *P *< 0.05. Protein spots that were differentially expressed in the hypoxia-treated and control groups (ratio_hypoxia/control _≥ 1.5 or ratio_hypoxia/control _≤ -1.5, *P *< 0.05) were marked.

During our studies, we ran three gels for each DIGE analysis on one batch of *C. elegans*, and we totally used three batches of *C. elegans *as independent experiments to meet the standard for biological replicates. Therefore, three biological replicated were included in our analyses to guarantee the statistical analysis for the samples, and the statistical values were averaged from multiple independent experiments.

### Protein identification by MALDI-TOF-TOF-MS

For protein identification, preparative gels were loaded with 500-1000 μg of unlabeled sample. The electrophoretic conditions were the same as for the 2D-DIGE. After staining with Coomassie Brilliant Blue, the protein spots of interest were excised and destained with a solution of 25 mM ammonium bicarbonate and 50% ACN. The gels were then dried completely by centrifugal lyophilization. An in-gel digestion was performed with 0.01 μg/μl trypsin (Promega) in 25 mM ammonium bicarbonate for 15 h at 37°C. The supernatants were collected, and the tryptic peptides were extracted from the gel sequentially with 5% TFA at 40°C for 1 h and with a solution of 2.5% TFA and 50% ACN at 30°C for 1 h. The extracts were pooled and dried completely by centrifugal lyophilization.

The peptide mixtures were redissolved in 0.5% TFA, and 1 μl of the peptide solution was mixed with 1 μl of matrix (4-hydroxy-α-cyanocinnamic acid in 30% ACN and 0.1% TFA) before spotting on the target plate. MALDI-TOF mass spectrometry and tandem TOF/TOF mass spectrometry were performed on a ABI-4800 Proteomics Analyzer. Peptide mass maps were acquired in positive reflection mode, averaging 1500 laser shots per MALDI-TOF spectrum and 3000 shots per TOF/TOF spectrum with a resolution of 20,000. The 4800 calibration mixtures were used to calibrate the spectrum to a mass tolerance within 0.1 Da. Parent mass peaks with a mass range of 600-4000 Da and minimum signal-to-noise ratio of 15 were selected for tandem TOF/TOF analysis. The combined mass and mass/mass spectra were used to interrogate *C. elegans *sequences in the Swiss-Prot database using the MASCOT database search algorithms, allowing for carbamidomethylation, oxidation, and a maximum of one missed trypsin cleavage. The peptide and MS/MS tolerances were both 0.2 Da. All of the automatic data analysis and database searches were performed by the GPS Explorer™ software (version 3.6, Applied Biosystems). Known contaminant ions (such as keratin) were excluded. A confident identification had a statistically significant (*P <*0.05) protein score (based on combined mass and mass/mass spectra) and a best ion score (based on mass/mass spectra).

### Western blot

Proteins from the hypoxia-treated *C. elegans *and control animals were separated on 12% polyacrylamide gels and transferred to PVDF membranes (Amersham Biosciences). These blots were incubated for 2 h at room temperature in Tris-buffered-saline with Tween (20 mM Tris-Cl, 140 mM NaCl, pH 7.5, 0.05% Tween 20) containing 5% skim milk. The blots were incubated with an anti-tropomyosin monoclonal antibody (working dilution 1:1000, Sigma-Aldrich) overnight at 4°C. After washing three times in Tris-buffered-saline with Tween, the blots were incubated with a horseradish peroxidase-conjugated secondary antibody (working dilution 1:5000, Beijing Zhong-Shan Biotechnology) for 1 h at room temperature. Immunoreactive complexes were visualized using ECL reagents (GE Healthcare). The housekeeping protein α-tubulin was used as an internal control (monoclonal antibody, working dilution 1:400, Wuhan Boshide Biotechnology). Protein expression levels were quantified with the ImageJ software. The data was statistically analyzed by OriginPro v8.0 software using a paired Student's *t*-test, with *P <*0.05 indicating a significant difference.

### Analysis of death rate after hypoxia

The *lev-11, daf-2 *(hypoxia-resistant*), daf-16 *(hypoxia-sensitive) mutant strains were kindly provided by the CGC and cultured as described above. Approximately 100 to 150 L4 larvae from each of the three mutant strains and the wild type (N2) were quickly harvested in a tube with M9 buffer, washed three times, and incubated for 8 h, 10 h,12 h, 14 h and 16 h in a sealed hypoxia chamber (0.2% oxygen, 26°C). The worms were then cultured at normoxia (21% oxygen, 20°C) on 3-cm nematode growth medium agar plates seeded with *E. coli *OP50. After 24 h, the numbers of living and dead worms were counted, which were used to calculate the death rate. Worms without spontaneous or touch-evoked movement were scored as dead. The experiments were repeated at least three times (n > 3). The statistical analysis was performed by OriginPro v8.0 software using a paired Student's *t*-test, with *P <*0.05 indicating a significant difference.

### Gene Ontology (GO) Analysis

Searches for the gene ontology (GO) classifications of the proteins identified by MALDI/TOF/TOF were performed using the web-accessible DAVID annotation system http://david.abcc.ncifcrf.gov/.

## Competing interests

The authors declare that they have no competing interests.

## Authors' contributions

HL wrote the main manuscript and did the most of the experiments. CR and JS helped on the *C. elegans *models for this study and completed the additional studies for revising the manuscript. XH helped on the bioinformatic analysis. FZ, YG and YW helped on the Western blot analysis. LX and CC gave English writing suggestions and statistical analyses on this work. CZ designed and supervised the study, finalized the manuscript. All authors read and approved the manuscript.
